# Computational Prediction of Alanine Scanning and Ligand Binding Energetics in G-Protein Coupled Receptors

**DOI:** 10.1371/journal.pcbi.1003585

**Published:** 2014-04-17

**Authors:** Lars Boukharta, Hugo Gutiérrez-de-Terán, Johan Åqvist

**Affiliations:** Department of Cell and Molecular Biology, Uppsala University, Biomedical Center, Uppsala, Sweden; University of Maryland, Baltimore, United States of America

## Abstract

Site-directed mutagenesis combined with binding affinity measurements is widely used to probe the nature of ligand interactions with GPCRs. Such experiments, as well as structure-activity relationships for series of ligands, are usually interpreted with computationally derived models of ligand binding modes. However, systematic approaches for accurate calculations of the corresponding binding free energies are still lacking. Here, we report a computational strategy to quantitatively predict the effects of alanine scanning and ligand modifications based on molecular dynamics free energy simulations. A smooth stepwise scheme for free energy perturbation calculations is derived and applied to a series of thirteen alanine mutations of the human neuropeptide Y1 receptor and series of eight analogous antagonists. The robustness and accuracy of the method enables univocal interpretation of existing mutagenesis and binding data. We show how these calculations can be used to validate structural models and demonstrate their ability to discriminate against suboptimal ones.

## Author Summary

G-protein coupled receptors constitute a family of drug targets of outstanding interest, with more than 30% of the marketed drugs targeting a GPCR. The combination of site-directed mutagenesis, biochemical experiments and computationally generated 3D structural models has traditionally been used to investigate these receptors. The increasing number of GPCR crystal structures now paves the way for detailed characterization of receptor-ligand interactions and energetics using advanced computer simulations. Here, we present an accurate computational scheme to predict and interpret the effects of alanine scanning experiments, based on molecular dynamics free energy simulations. We apply the technique to antagonist binding to the neuropeptide Y receptor Y1, the structure of which is still unknown. A structural model of a Y1-antagonist complex was derived and used as starting point for computational characterization of the effects on binding of alanine substitutions at thirteen different receptor positions. Further, we used the model and computational scheme to predict the binding of a series of seven antagonist analogs. The results are in excellent agreement with available experimental data and provide validation of both the methodology and structural models of the complexes.

## Introduction

G-protein coupled receptors (GPCRs) are an important group of membrane proteins that mediate physiological signals from the outside to the inside of cells. They are targets for approximately 30% of all prescribed drugs and of major interest to the pharmaceutical industry [1]. The understanding of GPCR structure, function and ligand binding has traditionally advanced through a combination of biochemical experiments and computationally generated 3D structure models [2]. Common experimental approaches include site-directed mutagenesis, generation of chimeric receptors and the substituted-cysteine accessibility method, while 3D models are used for design and interpretation of such experiments. In recent years, the field has benefitted enormously from breakthroughs in membrane protein crystallography, with a steadily increasing number of GPCR crystal structures determined since 2007 [3]. These structures not only enable structure-based drug design for crystallized targets but also make modelling of homologous GPCRs for the same purpose feasible [4]. Computational modelling is of optimal use in combination with site-directed mutagenesis data and structure-activity relationships for series of ligands [5], but requires careful validation.

Reliable free energy calculations based on molecular dynamics (MD) simulations can provide the missing links between experimental binding affinities and 3D structures of protein-ligand complexes [6]. In particular, approaches based on the free energy perturbation (FEP) method enable the evaluation of relative binding free energies between different ligands binding to a given receptor as well as to mutant versions of it [7], [8]. These techniques can yield accurate and convergent results provided that the complexes compared are not too dissimilar [9],[10]. However, when ligands differ by larger substituents, or receptors differ by more drastic mutations (e.g., tryptophan to alanine), the methodology becomes considerably less reliable due to convergence and sampling problems associated with the simulations. Hence, reliable FEP schemes for the systematic prediction of ligand binding and mutagenesis effects are rather scarce, and particularly so in the field of GPCRs where they would have a large impact [11]. The basic problem with applying free energy calculations to complexes that differ substantially in chemical structure is both that numerical instabilities can arise and that conformational sampling becomes more critical, when large groups of atoms vanish or appear during the computational “alchemical” transformations used [8]. To overcome this limitation, we present here a new FEP scheme for accurate calculation of the energetics of alanine scanning, which is applied to characterize the binding of antagonists to the human neuropeptide Y (NPY) receptor type 1 GPCR.

The NPY system is comprised in mammals by three neuronal and endocrine peptides (NPY, peptide YY and pancreatic polypeptide) which activate receptors belonging to the rhodopsin-like (class A) GPCRs. Four functional receptors named Y1, Y2, Y4 and Y5 exist in humans and are all expressed in the peripheral and central nervous system. The NPY system has broad biological functions, including involvement in control of feeding behavior, cortical neural activity and emotional regulation. As a consequence, this system has been implicated in several human diseases such as obesity, alcoholism and depression [12]. However, until now no effective drugs have been developed for the NPY system, an area that would definitely benefit from structural insights into receptor-ligand interactions. With no crystal structures yet determined for any of the Y receptors, homology modelling in combination with site-directed mutagenesis has proven extremely useful for characterization of receptor-ligand interactions [13].

BIBP3226 is a competitive and Y1-selective antagonist which is widely used as a pharmacological tool for studying the physiological role of the Y1 receptor. For therapeutic application, however, the compound has drawbacks with regard to toxicity as well as low oral availability and brain penetration [14]. There is extensive experimental data available in the literature for this particular receptor-ligand pair, with binding studies for BIBP3226 to both wild-type (wt) and alanine mutants of Y1 [15], [16], as well as Y1 wt binding data for numerous BIBP3226 analogs [17], [18]. We apply our new free energy perturbation scheme to a combined data set of alanine scanning for thirteen amino acids in the binding site region of Y1 and the binding of seven analogs of BIBP3226, and show how this methodology can be efficiently used to validate structural models of the hY1-BIBP3226 complex. The structural insights obtained further demonstrate the applicability of the approach in ligand design projects aimed at structure-based development of new GPCR ligands.

## Results

### GPCR modelling and structural stability

**Figure 1:**
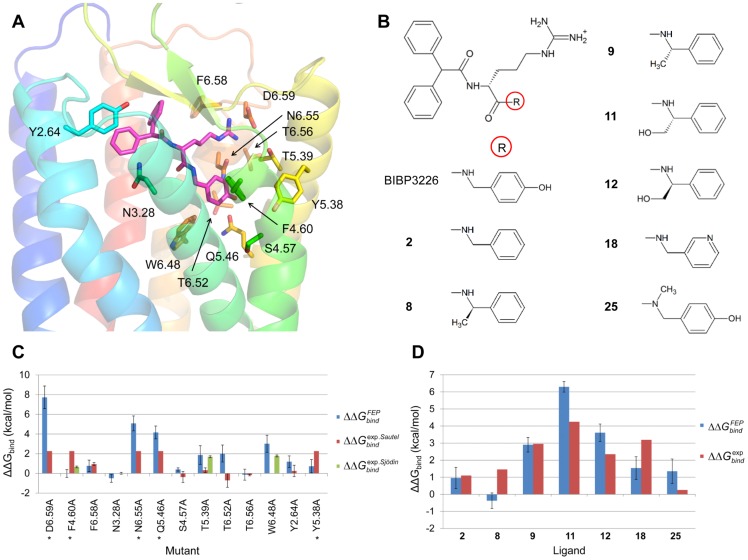
Structure of the hY1-BIBP3226 complex, ligand analogs and relative binding free energies. (A) Starting structure for the FEP calculations. The TM helices of hY1 are shown in anti-clockwise order (TM1, dark blue – TM7, red). Residues for which alanine scanning has been done are coloured according the TM helices and BIBP3226 is shown with magenta carbons. (B) Structure of BIBP3226 and seven analogs [17], [18], where the ligands differ in the R substituent. (C) Calculated and experimental relative binding free energies for BIBP3226 to the thirteen hY1 alanine mutants compared to hY1 wt. Blue bars represent 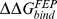, red bars 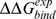 from Sautel *et al.* [15] and green bars 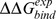 from Sjödin *et al.* ^16^. For mutants marked with an *, 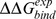 measured by Sautel *et al.* ^15^ is larger than 2.3 kcal/mol. (D) Calculated and experimental relative hY1 wt binding free energies for the seven compound analogs compared to BIBP3226. Blue bars represent 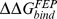 and red bars 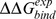 from Aiglstorfer *et al.* [17], [18]. Error bars are ±1 s.e.m.

In this work thirteen amino acids in the binding site region of Y1 are mutated to alanine using the free energy perturbation technique, namely Y2.64, N3.28, S4.57, F4.60, Y5.38, T5.39, Q5.46, W6.48, T6.52, N6.55, T6.56, F6.58 and D6.59 (Figure 1 and Table S1, Supporting Information). Experimental relative binding free energies for the hY1 mutants compared to the wt receptor were derived from BIBP3226 *K*_i_ values [15], [16], whereas relative binding free energies between the reference compound BIBP3226 and the seven analogs (Figure 1, Table S2) were estimated from experimental *IC*_50_ values [17], [18] for wt hY1 (Methods). The hY1-BIBP3226 complex that was used as starting structure for all FEP calculations is shown in Figure 1A. The system was generated by homology modelling of hY1 with the program Modeller [19], followed by insertion of the model in a lipid bilayer and refinement by MD equilibration using GROMACS4.0.5 [20], as implemented in the GPCR-ModSim web server [21]. Then both automated docking with Glide [22] and mutagenesis-guided docking of BIBP3226 into the hY1 model were carried out, and the resulting complexes were subject to a final round of MD equilibration using a spherical simulation system using the program Q [23], which also allows for very efficient FEP calculations [6]. Based both on structural stabilities of the wt hY1− BIBP3226 complexes and subsequent free energy calculations, the mutagenesis-guided docking approach was found to provide the best starting model (see below). In this complex BIBP3226 is positioned at the bottom of the hY1 orthosteric binding cavity. The deep pocket between F4.60 and W6.48 is occupied by the phenol moiety of BIBP3226, which places the hydroxyl group at hydrogen bond distance to both Q5.46 and N6.55. The guanidinium group of the ligand forms a salt bridge with the key NPY receptor residue D6.59 [15], [16], [24] and hydrogen bonds to N6.55. The pocket between transmembrane (TM) helices TM2, TM3 and TM7 and extracellular loop 2 accommodates the biphenyl moiety of BIBP3226.

The position of the ligands and their interactions with the receptors were generally very stable throughout the MD simulations. As an example, the BIBP3226 heavy atom RMSD was only 0.3 Å between the initial structure and the average wt structure from a total of (13+7)×6 = 120 independent equilibration runs (60 ns) for this complex. Analogously, the RMSD of the side chain heavy atoms belonging to the binding site (defined as all residues within 5 Å of the ligand) was also very low (RMSD = 0.5 Å). The only exceptions to this stability were two types of mutations. The first includes the N6.55A and D6.59 receptor mutations which both involve the deletion of a key polar interaction with the D-arginine moiety of BIBP3226, thereby rendering the ligand more flexible and shifting its position somewhat in the binding pocket. The second type is ligand modifications that remove the hydroxyl group from BIBP3226, which provides the hydrogen bonds responsible for attachment to both N6.55 and Q5.46.

### Free energy perturbation scheme

Free energy simulations of single point mutations where larger residues are mutated to alanine (alanine scanning) involve the annihilation of a substantial number of atoms. The conformational states of the native (wt) protein and a given alanine mutant are then often too dissimilar for standard FEP protocols to yield accurate and convergent results. The most common ways to computationally transform the protein from wt to mutant is either to simultaneously change both electrostatic and van der Waals interaction potentials or to do it separately in two stages. It has been established that in the annihilation of repulsive atomic centers, an intermediate stage with so-called soft-core potentials (that avoid singularities) is beneficial for convergence [25]. However, the main problem with these approaches is still that the transformation between each stage is carried out via linear combinations of the end state potentials for all atoms involved.

**Figure 2:**
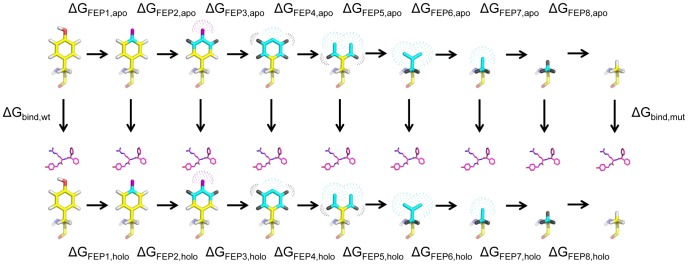
Thermodynamic cycle for a Tyr→Ala mutation. The transformation is divided into a series of smaller subperturbations involving additional intermediate states (horizontal paths). Yellow carbons, red oxygen and white hydrogens represent regular partial charge and van der Waals parameters. Cyan carbons, purple oxygen and black hydrogens represent atoms with zero partial charge. Dotted surfaces represent soft-core van der Waals parameters. The upper row corresponds to the apo state and the lower row to the holo state (with the presence of the ligand indicated). Calculated free energy values and their decomposition (vertical arrows) and given in Table 1.

**Figure 3:**
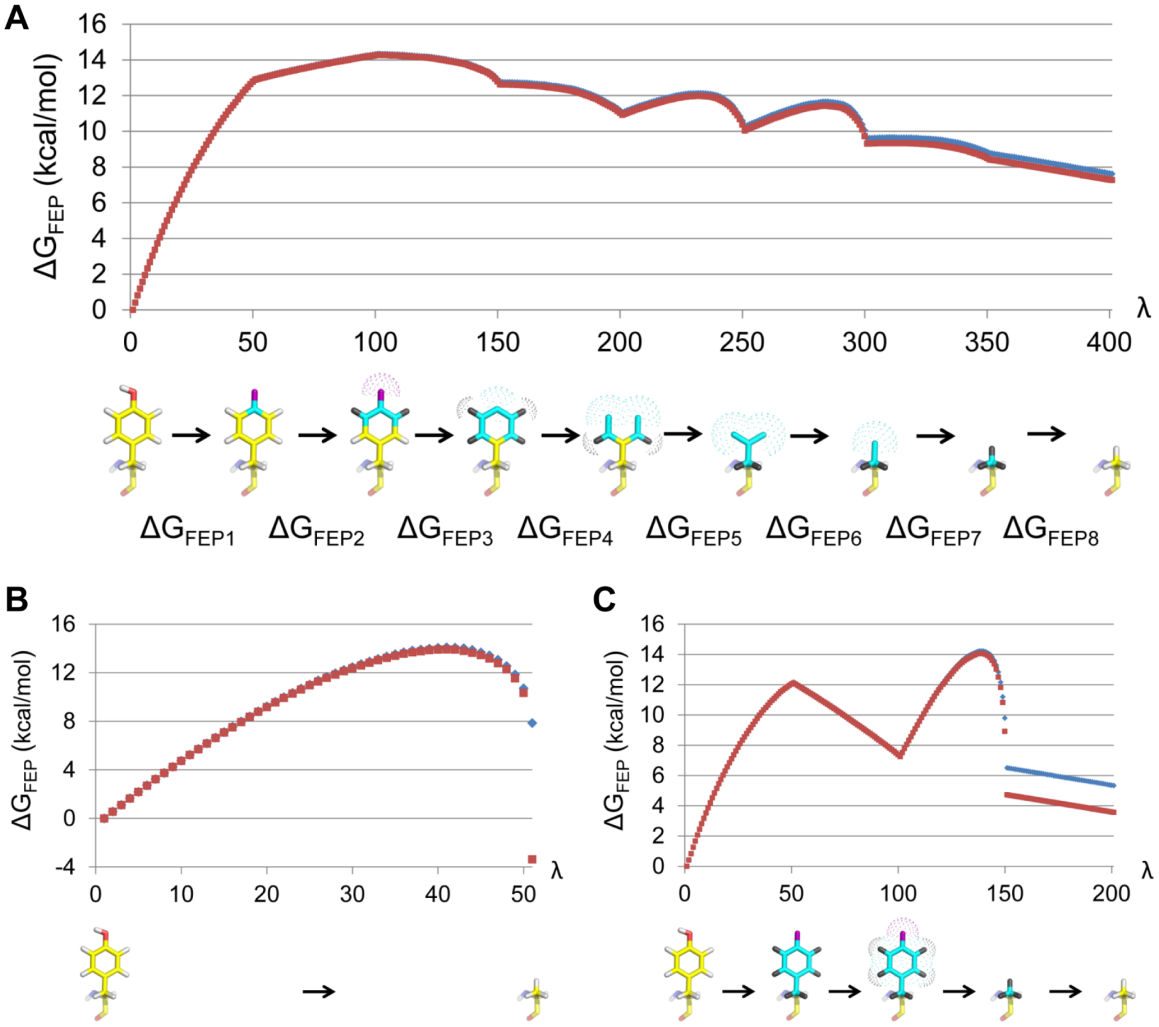
Free energy change for the Y2.64A mutation in the hY1 apo structure with different FEP protocols. Blue and red curves are averages over six independent simulations and correspond to application of the FEP formula in the forward (Tyr→Ala) and reverse (Ala→Tyr) directions, respectively. (A) The FEP scheme derived in this work, where the calculations correspond to the upper row of the thermodynamic cycle in Figure 2. 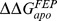 = 7.4±0.5 kcal/mol (error bar 1 s.e.m.) with a hysteresis error of 0.35 kcal/mol. (B) Result for the most basic reference FEP protocol. 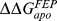 = 2.2±0.9 kcal/mol with a hysteresis error of 11 kcal/mol. (**c**) Result for the reference protocol utilizing soft-core potentials and separate transformation of electrostatics and van der Waals potentials, but applied to all atoms simultaneously. 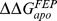 = 4.4±0.3 kcal/mol with a hysteresis error of 1.8 kcal/mol. The total simulation time is equal for all protocols.

To overcome this problem, we instead constructed a smooth scheme based on successive fragment annihilation, which is illustrated for the case of a Tyr→Ala mutation in Figure 2. The basic idea is to divide the whole transformation into a series of smaller “subperturbations” between a number of additional intermediate states, which are designed to be similar enough to ensure convergent free energy differences. Each subperturbation is as usual divided into a series of even finer grained FEP windows, yielding a total number of perturbation steps of several hundred (Figure 3). This strategy is not to be confused with the nowadays outdated “slow growth” method [26] in which only the two end states are used together with a transformation potential that changes in every MD step. In our scheme we defined groups of atoms in the wt residue (Figure 2 shows the Tyr example), based on their distance to the Cβ atom. Each group will undergo three consecutive types of transformations during its annihilation: charge annihilation, regular van der Waals (Lennard-Jones) potential transformation to soft-core and, finally, annihilation of the soft-core potential. In the Tyr→Ala case five atom groups are defined and eight independent subperturbations are used (Figure 2). For cases where new atoms are instead created, as in the BIBP3226 ligand perturbations discussed below, the scheme is simply reversed and annihilation and creation of groups can also, of course, be treated simultaneously.

We assessed the precision of our method for every protein and ligand mutation from six independent MD/FEP simulations, each corresponding to a total length of 4–6 ns including all subperturbations. Besides the precision, a critical convergence measure is the hysteresis resulting from applying the FEP formula (see Methods section) in the forward and reverse summation direction for each individual simulation. The average hysteresis obtained in this way from the six replicate trajectories for each alanine scan FEP calculation was in the range 0.0–0.5 kcal/mol, with an overall average for all mutations of 0.25 kcal/mol. The corresponding hysteresis range for the BIBP3226 ligand mutations was 0.0–0.1 kcal/mol, with an average over all ligands of 0.06 kcal/mol. These hysteresis errors are, in fact, remarkably small and clearly demonstrate the efficiency of our FEP scheme. As an illustration, Figure 3A shows the forward and reverse progression of the free energy change for a Tyr→Ala mutation in the hY1 apo structure corresponding to the upper row of the thermodynamic cycle in Figure 2. Furthermore, the precision of the different free energy calculations, in terms of standard errors of the mean (s.e.m.) based on the six independent trajectories, is very satisfactory and typically about 0.5 kcal/mol for the different protein simulations and ≤0.2 kcal/mol for the BIBP3226 mutations in water (Table 1 and Table S3).

**Table 1:** Calculated and experimental BIBP3226 relative binding free energies for wt and mutant hY1 receptors.^a^

Position	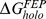	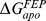	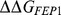	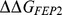	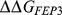	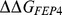	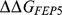	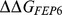	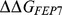	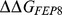	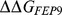	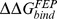	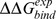 b
D6.59A	32.8±0.7	25.1±0.9	7.4±1.1	0.0±0.1	0.2±0.2	0.2±0.1	−0.1±0.0					7.7±1.2	>2.3
F4.60A	0.6±0.3	0.6±0.2	0.0±0.0	0.0±0.2	−0.2±0.0	−0.2±0.1	0.2±0.2	0.1±0.2	0.0±0.3	0.0±0.0		0.0±0.4	>2.3
													0.7±0.1^c^
F6.58A	−1.1±0.4	−1.8±0.4	0.1±0.1	0.6±0.2	−0.1±0.1	0.1±0.1	0.4±0.4	−0.1±0.2	−0.3±0.2	0.0±0.0		0.8±0.6	1.0±0.1
N3.28A	39.5±0.3	40.0±0.3	−0.1±0.3	−0.2±0.0	−0.1±0.1	−0.2±0.2	0.1±0.0					−0.5±0.4	0.0±0.1^c^
N6.55A	45.0±0.7	39.9±0.3	5.5±0.7	−0.3±0.2	0.0±0.3	−0.1±0.4	0.0±0.0					5.1±0.8	>2.3
Q5.46A	41.4±0.6	37.3±0.3	3.3±0.5	−0.1±0.1	0.2±0.3	0.7±0.4	0.0±0.1	0.1±0.0				4.2±0.7	>2.3
S4.57A	−0.7±0.1	−1.1±0.1	0.5±0.1	0.0±0.1	−0.1±0.1	0.0±0.0						0.4±0.2	−0.4±0.6
T5.39A	−2.0±0.8	−3.9±0.6	1.4±1.0	−0.1±0.0	0.3±0.2	0.3±0.2	0.0±0.0					1.9±0.9	0.3±0.2
													1.7±0.1^c^
T6.52A	−5.2±0.4	−7.2±0.8	2.3±0.9	−0.1±0.0	−0.3±0.1	0.1±0.1	0.0±0.0					2.0±0.9	−0.7±0.7
T6.56A	−4.0±0.3	−3.9±0.5	−0.3±0.1	0.2±0.1	0.2±0.4	−0.1±0.2	−0.1±0.0					−0.1±0.6	−0.2±0.1
W6.48A	13.5±0.6	10.5±0.7	0.2±0.0	0.3±0.1	0.1±0.0	1.2±0.4	0.4±0.3	0.7±0.2	0.2±0.4	−0.3±0.3	0.0±0.0	3.0±0.9	1.8±0.1^c^
Y2.64A	8.6±0.2	7.4±0.5	0.0±0.2	0.1±0.1	0.9±0.2	0.3±0.2	−0.7±0.2	0.4±0.2	0.0±0.1	0.0±0.0		1.2±0.6	0.3±0.6
Y5.38A	10.5±0.4	9.8±0.5	0.4±0.1	−0.1±0.1	−0.3±0.1	0.1±0.1	0.3±0.4	0.2±0.5	0.1±0.2	0.0±0.0		0.7±0.7	>2.3

a The experimental values are derived from *K*_i_ values [15], [16]. Calculated energies 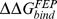 are obtained using a series of small, convergent FEP calculations (Δ*G_FEP{X},holo_* and Δ*G_FEP{X},apo_*) and expressed in kcal/mol.

b Experimental data from Sautel *et al.* [15] except

c data from Sjödin *et al.* [16].

**Figure 4:**
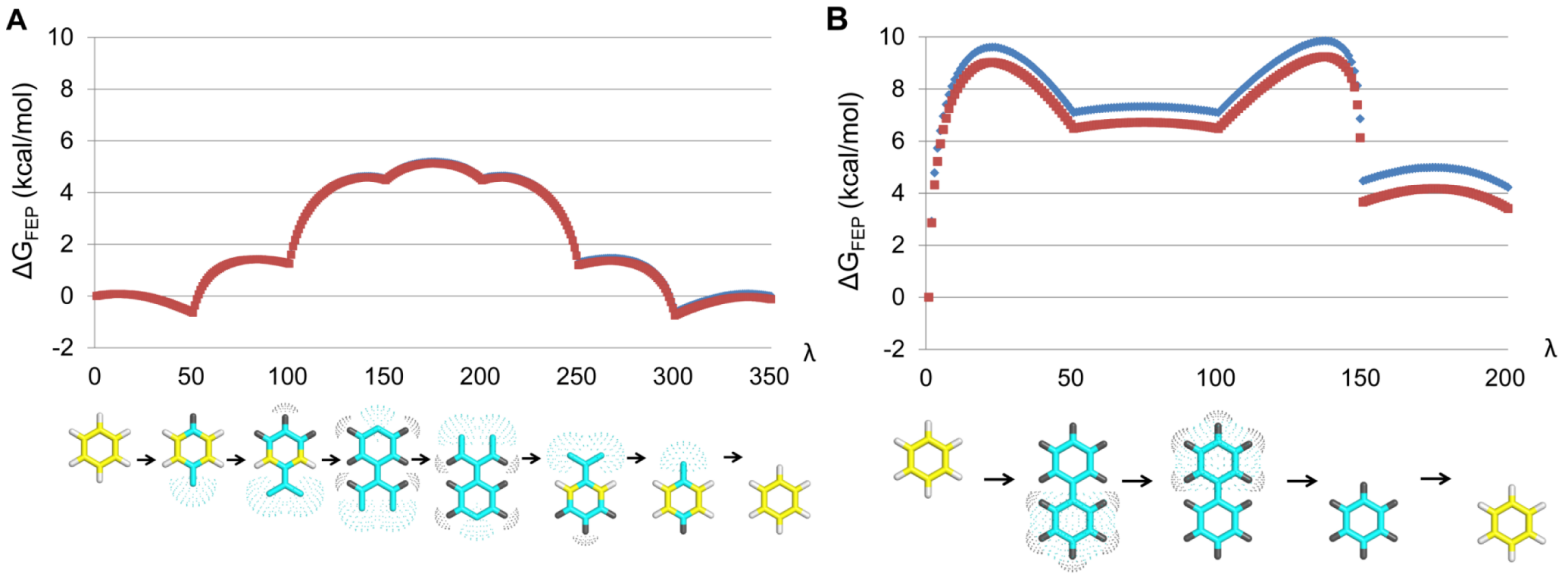
Free energy change for a phenyl to phenyl group null transformation with two different FEP protocols. The correct Δ*G* between the two states is exactly zero. Blue and red curves as in Figure 3 based on ten independent simulations. (A) Result for the main protocol derived in this work. Δ*G* = −0.06±0.07 kcal/mol (error bar 1 s.e.m.) with a hysteresis error of 0.13 kcal/mol. (B) Result for the reference protocol. Δ*G* = 3.8±0.2 kcal/mol with a hysteresis error of 0.4 kcal/mol. The total simulation time is equal for both protocols.

The above results can be compared to those of less intricate reference protocols as shown in Figure 3. The first of these (Figure 3B) transforms electrostatic and van der Waals parameters simultaneously with no extra intermediate states. The second reference scheme utilizes intermediate soft-core [25] van der Waals interactions and separate transformations of electrostatic and van der Waals potentials, but performs the operations on the entire sidechain simultaneously (Figure 3C). Intermediate states with soft-core potentials clearly reduce the hysteresis error to some extent (Figure 3C), but it is evident that the stepwise elimination of atoms, with many extra intermediate states, is key to the superior performance of our method (Figure 3A). As an additional control, Figure 4 shows analogous FEP curves for our scheme and the second reference protocol, extracted from a transformation where one phenyl group is created and one simultaneously annihilated in water. This is a useful benchmark since the correct free energy change is exactly zero and both hysteresis errors and accuracy (in this case based on ten independent simulations) can be assessed. The result of the FEP calculations utilizing our new method is Δ*G* = −0.06±0.07 kcal/mol with an average hysteresis error of 0.13 kcal/mol (Figure 4A). Hence, convergence (hysteresis), precision and accuracy are all excellent. In contrast, the performance of the reference protocol is considerably worse with Δ*G* = 3.8±0.2 kcal/mol with a hysteresis of 0.4 kcal/mol (Figure 4B).

### Computational alanine scanning results

The relative binding free energies calculated from the MD/FEP simulations are generally in good agreement with experimental values, thus supporting the validity of the underlying structural model. For the alanine mutations the mean unsigned error with respect to experimental BIBP3226 binding free energies is 0.9 kcal/mol and the method is generally successful in discriminating mutations that have large effects on ligand binding from those that have only minor effects (Figure 1C). If only the data from Sjödin et al. is considered, which has smaller relative experimental errors [16], the performance of the FEP calculations improves (<|error|> = 0.6 kcal/mol) and better agreement is observed in this case for the two independently measured mutations [15], [16] F4.60A and T5.39A (Figure 1C). Moreover, for the six mutations for which 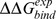 has been determined with an uncertainty of less than 0.2 kcal/mol, the mean unsigned error of the calculations is only 0.5 kcal/mol (Table 1).

Comparison of binding free energy differences between calculations and experiment can thus be used to validate the structural model. Here, the agreement is very good in most instances indicating that this GPCR-antagonist model has a close resemblance to the correct structure. The binding pocket between TM3, TM4, TM5 and TM6 and its interactions with the 4-hydroxybenzylamine and D-arginine groups of BIBP3226 are the part of the structure that is most thoroughly validated. In our structure, six of the thirteen mutated amino acids - F4.60, T5.39, Q5.46, W6.48, N6.55 and D6.59 - line the wall of this subpocket and the ligands differ only in this region (Figure 1A). The FEP calculations reproduce the large positive ΔΔ*G_bind_* associated with mutating D6.59, N6.55 and Q5.46 to alanine (Figure 1C). In the hY1 structure these three residues have ionic and polar interactions with the guanidinium and hydroxyl groups of the ligand (Figure 1A). It can be clearly seen from the FEP calculations that the large ΔΔ*G_bind_* is primarily due to considerably more favourable electrostatics for the D6.59, N6.55 and Q5.46 sidechains in the holo structure compared to the apo structure (ΔΔ*G_FEP1_* in Table 1). Further, the large effect of the W6.48A mutation is also well reproduced by the simulations. When this tryptophan residue is mutated to alanine a cavity is created deep in the binding site and gradually filled with water, with the total change in binding free energy accumulating gradually over the series of smaller perturbations (Table 1). As mentioned, the experimental data for the two mutants F4.60A and T5.39A is ambiguous. One report indicates that F4.60 has a significant effect on BIBP3226 binding but that T5.39A has a negligible effect [15]. In contrast, the higher precision data say the opposite [16] which is also supported by the present FEP calculations (Figure 1C). In the structural model of the hY1 complex both of these residues are in contact with the ligand.

Residues Y2.64 and N3.28 face another part of the binding cavity, namely the pocket between TM2, TM3 and TM7 (Figure 1A). Y2.64 contacts one of the phenyl groups of the ligand and the FEP calculations yield a lower binding affinity for Y2.64A to BIBP3226 in accordance with experimental measurements. N3.28, on the other hand, is not in direct contact with the ligand and the calculations in this case predict no change in affinity of N3.28A for the antagonist, again in agreement with experiment. The five remaining mutated residues are situated in interfaces between TM helices. Among these, S4.57A, T6.52A and T6.56A were shown in the experimental assays to bind BIBP3226 with essentially wt affinity [15]. The FEP calculations reproduce this pattern for S5.47A and T6.56A, while the binding free energy difference for T6.52A is overpredicted by 2.7 kcal/mol (Figure 1C). This is the only real outlier among the 13 alanine mutations examined, which might indicate that the conformation of this sidechain and/or its interaction network is not properly modeled. Finally, the calculations also reproduce the detrimental effect on BIBP3226 binding affinity for alanine mutations of the two aromatic residues F6.58 and Y5.38.

### Relative binding free energies between different ligands

The overall results of the simulations for the relative binding free energies of the BIBP3226 ligand series are remarkably good, with a mean unsigned error of 1.2 kcal/mol. Moreover, the method is clearly successful in discriminating the best binders from the low affinity ligands (Figure 1D). The calculations closely reproduce the weaker affinity of the dehydroxylated analog (**2**) as well as the larger effect of the combined dehydroxylated and (*S*)-methylated compound (**9**). Although ΔΔ*G_bind_* for the (*R*)-enantiomer of the latter compound (**8**) is somewhat underestimated by the FEP simulations, it is noteworthy that the structural model still correctly discriminates between the two enantiomers (**8** *vs.* **9**). Furthermore, the enantiomeric compounds **11** and **12**, which differ in the stereochemistry of their hydroxymethyl substituent at the same chiral center, are both correctly ranked and predicted to be low affinity ligands, in agreement with the experimental binding data. From the FEP calculations it is also clear that the low affinity of the hydroxymethyl compounds **11** and **12** is due to unfavorable desolvation in the hY1 binding pocket (see corresponding ΔΔ*G_FEP4_* values in Table S3). The calculations further yield diminished affinities for both the pyridine analog (**18**) and the tertiary amide compound (**25**).

### Control simulations with an erroneous initial structure

As a useful control of the ability of the free energy calculations to discriminate against suboptimal structural models, all of the above FEP simulations were also carried out for the top-ranked solution resulting from the automated docking of BIBP3226 to the hY1 model (Figure S1). This docking solution essentially has the ligand rotated 180° around its arginine sidechain thereby interchanging the binding cavities for the phenol and biphenyl groups. The conformation is intuitively unrealistic since it places the biphenyl moiety in the vicinity of a number of polar groups. With this ligand orientation the correlation with the experimental binding data for the series of analogs is completely lost, indicating that the substituted phenol moiety must be in the wrong place. Also the alanine scanning results deteriorate although the effect is not as pronounced, probably due to the fact that the ligand is still occupying the same cavities even though it is flipped. It is, however, noteworthy that both the N6.55A and Q5.46A mutations now become outliers, most likely because the hydrogen bonding interactions with the phenol have been lost. Although the prediction for T6.52A mutation is actually better for this model this probably just reflects our suspicion that this receptor sidechain is in the wrong conformation, as discussed above.

## Discussion

Thermodynamic cycle free energy perturbation methods, or alchemical free energy calculations as they are sometimes called, have been around for quite some time [27] and were early applied to biochemical problems such as ligand binding [28], [29], protein stability [30] and enzyme catalysis [31]. These applications were clearly of more exploratory character and it is only recently that more systematic use of the FEP technique has been made, particularly in studies of aqueous solvation [32], [33], but also for ligand design purposes [34] and other key biochemical problems dealing with molecular recognition [6]. However, reliable computational schemes for systematically quantifying the effects of protein mutations on ligand binding have largely been lacking. In particular, the feasibility of carrying out larger scale computational alanine scanning simulations would be of great importance in connection with such mutagenesis experiments, as these are one of the major experimental routes for probing protein-ligand interactions in the absence of 3D structures. This is especially true for membrane protein interactions with ligands, such as ion channel blocking and ligand binding to GPCRs, given the limited availability of structural information for these systems.

The free energy calculation scheme developed here turns out to be very efficient for systematically modelling the effect of single-point alanine mutations on protein-ligand binding, even for the complex case of a membrane receptor. The smooth stepwise transformation procedure overcomes the long-standing convergence problem with FEP simulations that involve the creation or annihilation of many atoms [9], [10]. When applied to the hY1-BIBP3226 system, the agreement between calculated and experimental binding free energies is remarkably good for the thirteen alanine mutations and the series of eight receptor antagonists. These results thus serve to validate the 3D model of the complex and, conversely, a severely erroneous model could immediately be identified as such based on the loss of correlation between calculations and experiment. It is also noteworthy that even for the most complex Trp→Ala mutation, which involves the annihilation of a complete indole ring, a precision within 1 kcal/mol can be attained with only about 35 ns simulation time for each of the holo and apo states. A key aspect with regard to efficiency when dealing with many mutants and/or ligand molecules is also the size of the simulation system. Hence, while the common practice in MD studies of membrane proteins is to set up large simulation systems encompassing lipid bilayer patches with lateral dimensions of a hundred Å or more and a large number of solvent molecules [11], [35] it is not clear that this strategy is optimal for doing many independent free energy calculations. After all, the goal in this case is not to simulate conformational changes distal to the binding site but to obtain as reliable free energy estimates as possible at a computational cost that allows many mutants or ligands to be evaluated. In this respect, reduced models that still yield correct local structural fluctuations of the binding site [36] may be significantly more efficient than larger scale models, precisely because they do not sample large scale conformational motions that require much longer timescales for convergence. A case in point here is large ribosome complexes where reduced models allow for extensive free energy calculations [6] at a low computational cost.

As far as GPCRs are concerned there has been considerable recent progress with virtual screening strategies using homology models, as exemplified by the D3 dopamine [37] and A_2A_ adenosine [38] receptors. These cases seem particularly favorable in terms of availability of experimental data. The D3 receptor both has structural templates with high homology and the existence of well-defined dopamine anchoring points, which is true for aminergic receptors in general. The A_2A_ homology model, on the other hand, was validated using a unique proprietary technology to generate and characterize hundreds of mutants in vitro [5], together with large amounts of available binding data. For systems that are structurally less well characterized it is questionable to what extent virtual screening based on docking to homology models is really meaningful. In this respect, the combination of experimental and computational alanine scanning, as well as free energy calculations of structure-activity relationships for a series of ligands, can provide the necessary validation needed for model refinement and subsequent virtual screening efforts. We have shown here that a computationally derived model of the Y1-antagonist complex, obtained from homology modeling and docking simulations, rationalizes the existing mutagenesis and binding data while a suboptimal model of the same complex clearly fails to do so.

## Methods

### Experimental binding affinities

Experimental relative binding free energies for the hY1 mutants compared to wt hY1 were derived from BIBP3226 *K*_i_ values as 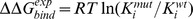. For the F4.60A and T5.39A mutants there are two sets of experimental values available from independent reports [15], [16], resulting in 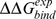 values that differ by at least 1.4 kcal/mol between the two sources. In these cases we used the average of the two measurements to assess the errors between the calculations and experiment. Further, mutations that have a BIBP3226 *K*_i_ value outside the concentration interval screened in the binding assay were not considered when calculating mean unsigned errors. Relative wt hY1 binding free energies between the reference compound BIBP3226 and the seven analogs were estimated from experimental *IC*_50_ values [17], [18] as 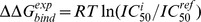.

### Homology modeling

The sequence of the hY1 receptor (Swiss-Prot accession number: P25929) was aligned with a multiple sequence alignment of all the inactive-like GPCRs of known structure using the GPCR-ModSim (http://gpcr.usc.es) web-server [39]. The human C-X-C chemokine receptor type 4 (hCXCR4) was considered the best template for modeling of the hY1 receptor because it is a peptide binding GPCR with high homology to hY1 in the C-terminal part of extracellular loop 2. This loop segment (Cys5.25-Ser5.31 in hY1) constitutes part of the orthosteric binding cavity wall and is often involved in ligand binding. Further, the hCXCR4 structures are determined in the inactive state in complex with antagonists [40]. This is important since BIBP3226 binds inactive state hY1. The sequence identity between hCXCR4 and hY1 in the transmembrane region is 29%. A chimeric template receptor was assembled making use of the structural alignment of the X-ray structures available from GPCR-ModSim. The chimeric template consisted mainly of hCXCR4 in complex with a cyclic peptide antagonist [40] (PDB entry 3OE0), but with some poorly defined intracellular parts extracted from two alternative templates: the intracellular loop 1 and the N-terminal end of TM6 from the hCXCR4 structure in complex with a small antagonist [40] (PDB entry 3ODU) while TM8 and the C-terminal end of TM7 were adopted from the hA_2A_R in complex with ZM241385 [41] (PDB entry 3EML). This chimeric structure was used as template for homology modeling of the hY1 receptor using the program Modeller 9.9 [19]. The hY1-hCXCR4 sequence alignment was manually refined in the longer loop regions and the N-terminus was discarded from hY1 modeling due to lack of sequence similarity. Five hundred homology models of the hY1 receptor were generated and the best candidate model was selected on the basis of low DOPE-HR assessment score [42] and orientation of Asp6.59 towards the binding crevice, a residue shown by mutagenesis to be important for both agonist and antagonist binding [15], [16], [24].

### Simulation system preparation and docking of BIBP3226

The hY1 model was treated with the membrane insertion and equilibration protocol implemented in the GPCR-ModSim web-server [21] (Figure S2A). Briefly, the system is embedded in a pre-equilibrated POPC (1- palmitoyl-2-oleoyl phosphatidylcholine) membrane model so that the TM bundle is parallel to the vertical axis of the membrane. The system is then soaked with bulk water and inserted into a hexagonal prism-shaped box of dimensions 118×121×100 Å, consisting of slightly more than 60.000 atoms. The system is energy minimized and equilibrated for 5 ns in a MD simulation with periodic boundary conditions (PBC) using GROMACS4.0.5 [20]. In the equilibration, a first phase of 2.5 ns where positional restraints for the protein atoms are gradually released is followed by 2.5 ns where positional restraints are only applied to the α-carbons [43]. The OPLS all-atom (OPLS-AA) force-field [44] was used with Berger united-atom parameters for the POPC lipids [45].

The binding mode of the antagonist BIBP3226 in the equilibrated homology model of hY1 was explored with two alternative docking strategies. First, automated docking with Glide SP (Glide, version 5.7, Schrödinger, LLC, New York, NY, 2011) was carried out, using default settings and a grid dimension of 30 Å×30 Å×30 Å centered on a point in the binding cavity halfway between T2.61 and S5.39, where the top ranked binding mode by GlideScore [22] was selected. Second, mutagenesis-guided docking was performed with PyMOL (Version 1.4.1, Schrödinger LCC, New York), using the extensive mutagenesis and structure-activity relationship data available [15]–[18] to guide placement of the ligand in the binding site. Here, we particularly required a salt bridge between D6.59 and the D-arginine moiety of BIBP3226 as well as hydrogen bonds between the ligand and the two residues Q5.46 and N6.55, as the experimental data indicate these interactions to be important. Briefly, the manual docking started from a lower ranked docking solution from Glide which had these polar contacts with the receptor. Manual adjustments of torsion angles and translation displacement of the ligand were performed in PyMOL to enhance the hydrogen bonds. The structural stability of the obtained ligand-receptor complex was evaluated using the MD equilibration protocol described below. The final mutagenesis-guided docking pose was generated after two iterative rounds of MD and manual adjustments. BIBP3226 binding modes from both strategies were further evaluated using MD and FEP calculations.

The hY1-BIBP3226 system was further equilibrated using the MD software Q [23]. A 40 Å radius spherical system was used, containing the predicted receptor-ligand complex with surrounding lipids and water molecules extracted from the equilibrated PBC simulation system described above (Figure S2B). Water molecules with oxygen atoms within 2.6 Å of any ligand heavy atom were removed. This spherical GPCR system was equilibrated for 2.1 ns using the MD settings described in detail below. From the final structure of this equilibration a 24 Å radius spherical simulation system was extracted and used as starting structure for all free energy calculations.

### MD simulations

MD simulations were carried out using Q with the OPLS-AA force-field [44]. Simulations of the holo and apo states of the hY1 receptor as well as free BIBP3226 in water were conducted with spherical systems with a radius of 24 Å (Figure S2C). The GPCR simulation systems were centered on a point in the orthosteric binding site situated approximately between T2.61 and T5.39. Ionizable residues near the edge of the spherical system were neutralized to avoid artifacts due to missing dielectric screening [46] and each system was solvated with TIP3P water [47]. For the holo state of hY1 the water configuration from the end point of the homology model equilibration was used as solvent starting structure. The starting structure for the apo state of hY1 was generated from the holo structure by replacement of BIBP3226 with water molecules. The free state of BIBP3226 was generated by solvation of the ligand with a 24 Å radius spherical water grid.

For the solvated GPCR systems, all atoms outside the 24 Å sphere were tightly restrained to their initial coordinates and excluded from non-bonded interactions. Further, a restraint of 10 kcal mol^−1^ Å^−2^ to the initial coordinates was applied to solute atoms within the outer 3 Å shell of the spherical systems. Water molecules at the sphere surface were subjected to radial and polarization restraints according to the SCAAS model [23], [48]. For the free ligand in water, a weak harmonic restraint was applied to the geometrical center of the solute to prevent it from approaching the sphere edge. The SHAKE algorithm [49] was applied to constrain solvent bonds and angles. A direct non-bonded interaction cutoff of 10 Å was used for all atoms except those that undergo parameter changes during the FEP calculation (for which no cutoff was applied), and long-range electrostatic interactions beyond the cutoff were treated with the local reaction field approximation [50]. In all simulations the system was slowly heated from 1 to 298 K while restraints on the solute coordinates to their initial position were gradually released. This was followed by 0.5 ns of unrestrained equilibration and 4–6 ns of FEP data collection (simulation time depending on the number of subperturbations) at 298 K, using an MD time step of 1 fs.

### Free energy perturbation calculations

In our FEP scheme, we divide the whole transformation into a series of smaller subperturbations between additional intermediate states, which are designed to be similar enough to ensure convergent free energy calculations. Each subperturbation is as usual divided into a series of even finer grained FEP windows, yielding a total number of perturbation steps of several hundred. The free energy difference associated with each subperturbation was calculated using Zwanzig's exponential formula [51]

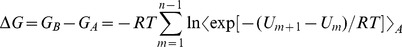
(1)where *U*_m_ denotes the effective potential energy function of a particular FEP window and *n* is the number of intermediate states. *U*_m_ is constructed as a linear combination of the initial (A) and final (B) potentials of the subperturbation
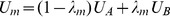
(2)where the coupling parameter 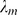 is stepwise incremented from 0 to 1. The subperturbations are defined by grouping atoms are based on their distance (number of bonds) to a fragment common to both the start and end state of the overall transformation. In the case of alanine scanning, the groups are thus defined by the distance to the Cβ atom. The annihilation of groups involve intermediate transformations of the regular van der Waals (Lennard-Jones) potentials transformation to soft-core interactions ^25^ which are given in Q [23] as
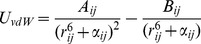
(3)where *A_ij_* and *B_ij_* are the Lennard-Jones parameters for the interaction between atoms *i* and *j*, *r_ij_* the distance between them and *α_ij_* is a constant that is set here to yield an energy of 20 kcal/mol at *r_ij_* = 0. The special case of D6.59A, which involves deletion of a charged sidechain, was treated by simultaneous charging of a chloride ion inside the water droplet about 20 Å from the position of the BIBP3226 positive charge in the holo structure (ΔΔ*G_FEP1_* in Table 1). As a test of this procedure, an alternative strategy where the missing ligand positive charge in the apo simulations was compensated by a K^+^ ion (thereby yielding the same net charge in the holo and apo states) was also examined. This gave an essentially identical result for D6.59A (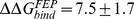 kcal/mol) but with a slightly lower precision.

Each subperturbation comprised 51 intermediate 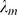 steps and at each step the system was simulated for 10–30 ps. Potential energies were collected every 21 fs and the first 1 ps of sampling in each state was discarded for equilibration. With eight subperturbations for the Tyr→Ala mutation (Figure 2), the total calculation thus involves about 400 intermediate states and a total data collection MD simulation of 5.6 ns. Six replicate FEP MD simulations with different initial atomic velocities were conducted for each mutation, where the initial state was the wt hY1 complex with BIBP3226. The relative binding free energy for each calculation is taken as an average of applying the FEP formula (eq. 1) in the forward and reverse directions, and all errors are reported as standard errors of the mean (s.e.m.). The hysteresis of a FEP calculation is defined here, for the whole transformation, as
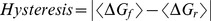
(4)Here, 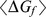 and 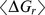 denote averages over the six independent simulations for applying eq. 1 in the forward and reverse summation directions, respectively. The total hysteresis is thus accumulated as the sum of the hysteresis associated with each subperturbation involved in the entire transformation.

In addition to the FEP calculations described above and in Figure 2, reference calculations were performed for Y2.64A in the hY1 apo structure using two less intricate FEP protocols. In the first control protocol Tyr was transformed into Ala using 49 intermediate states. Electrostatic and van der Waals parameters were altered simultaneously and no soft-core van der Waals potentials were utilized. The second control protocol consisted of a series of four FEP calculations using 199 intermediate states between Tyr and Ala. First, electrostatic parameters were changed to zero for all charge groups containing atoms to be annihilated. Second, van der Waals parameters were changed to soft-core van der Waals for all atoms not present in the end state. Third, the soft-core parameters were changed to the van der Waals parameters of the end state, which included annihilation of several atoms. Fourth, electrostatic parameters were changed from zero to the parameters of the end state. The number of replicate simulations, total MD simulation time and all settings were equal in all protocols. To further benchmark our FEP scheme, the phenyl to phenyl transformations of Figure 4 were performed utilizing both the main protocol and the second reference protocol described above. The MD simulations was carried out using the same settings as for the free ligands in water, with the exception that the phenyl molecule was solvated with a 18 Å radius spherical water grid. Ten replicate MD simulations of 3.57 ns each were conducted for both protocols.

## Supporting Information

Figure S1
**Structure of hY1-BIBP3226 complex generated with automated docking and calculated** ***vs*** **experimental relative binding free energies.** (A) Starting structure for these negative control FEP calculations with colouring as in Figure 1. (B) Calculated and experimental relative hY1 wt binding free energies for the seven compound analogs compared to BIBP3226. Blue bars represent 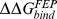 and red bars 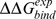 from Aiglstorfer *et al.* [17], [18]. (C) Calculated and experimental relative binding free energies for BIBP3226 to the thirteen hY1 alanine mutants compared to hY1 wt. Blue bars represent 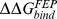, red bars 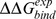 from Sautel *et al.* [15] and green bars 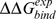 from Sjödin *et al.* [16]. For mutants marked with an *, 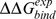 measured by Sautel *et al.* [15] is larger than 2.3 kcal/mol. Error bars are ±1 s.e.m.(TIF)

Figure S2 **Schematic view of the three-step setup for the MD equilibration and production phases.** (A) Starting model of the GPCR embedded in a lipid bilayer and simulated with PBC. (B) Equilibration of a 40 Å radius sphere centered on the ligand binding site that was cut out from the larger system. (C) The reduced model of the receptor-membrane-water system used for FEP calculations, where the radius of the simulation sphere is decreased to 24 Å.
(TIF)

Table S1 ***K_i_*** **values for BIBP3226 binding to wt and mutant hY1 from two different sources** [**15]**, [**16**] **.**
(DOCX)

Table S2 **Structure and hY1 antagonistic activity of BIBP3226 analogs** [**17]**, [**18**] **.**
(DOCX)

Table S3 **Calculated and experimental relative binding free energies of BIBP3226 analogs to wt hY1.**
(DOCX)
