# Supplementary figures and images for: Computational Prediction of Alanine Scanning and Ligand Binding Energetics in G-Protein Coupled Receptors
Source: PLoS Comput Biol. 2014 Apr 17;10(4):e1003585. doi: 10.1371/journal.pcbi.1003585 (PMC3990513; doi:10.1371/journal.pcbi.1003585)

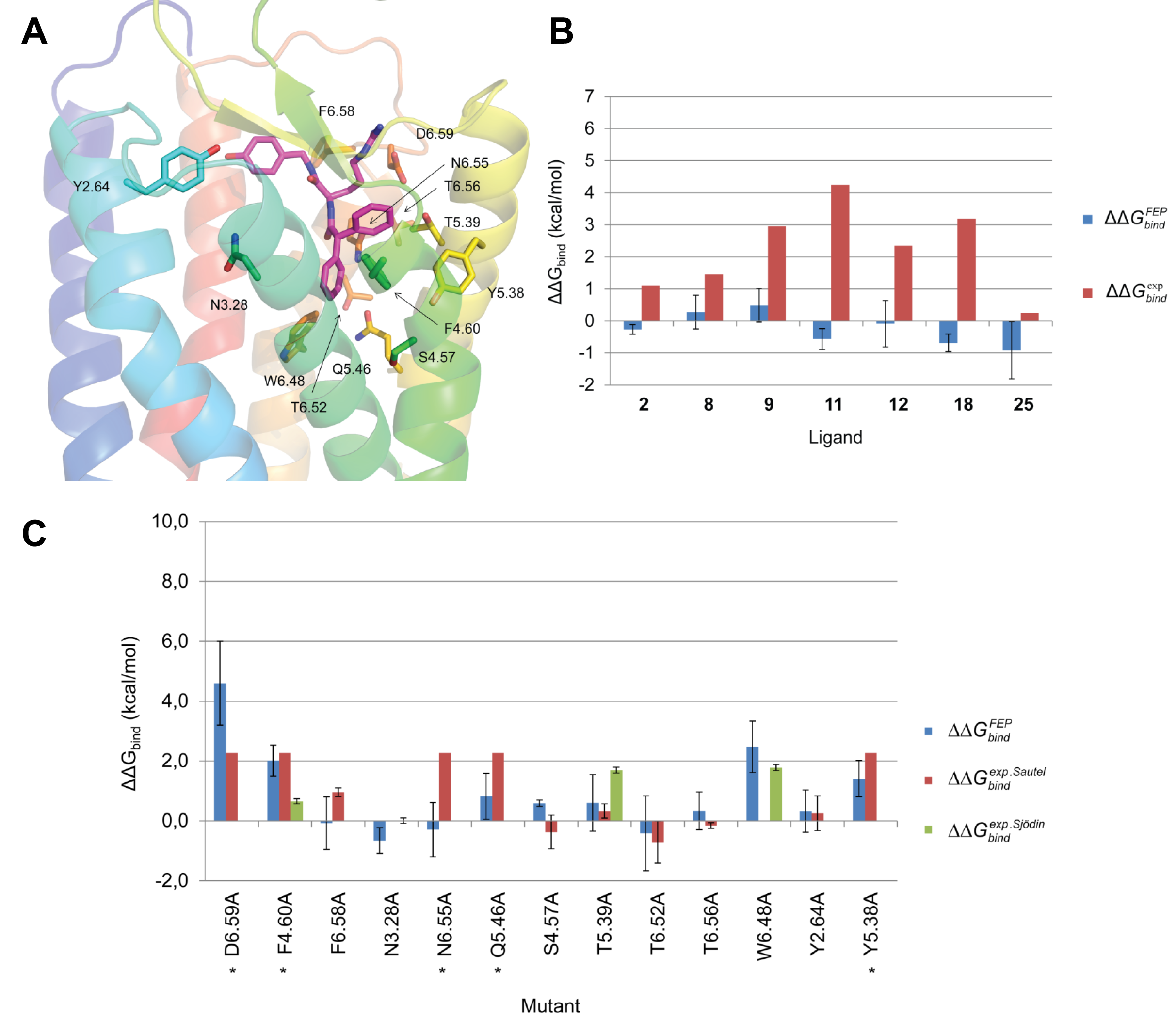

Supplement: Figure S1 — Structure of hY1-BIBP3226 complex generated with automated docking and calculated vs experimental relative binding free energies. (A) Starting structure for these negative control FEP calculations with colouring as in Figure 1. (B) Calculated and experimental relative hY1 wt binding free energies for the seven compound analogs compared to BIBP3226. Blue bars represent and red bars from Aiglstorfer et al. [17], [18]. (C) Calculated and experimental relative binding free energies for BIBP3226 to the thirteen hY1 alanine mutants compared to hY1 wt. Blue bars represent , red bars from Sautel et al. [15] and green bars from Sjödin et al. [16]. For mutants marked with an *, measured by Sautel et al. [15] is larger than 2.3 kcal/mol. Error bars are ±1 s.e.m. (TIF) [file pcbi.1003585.s001.tif]

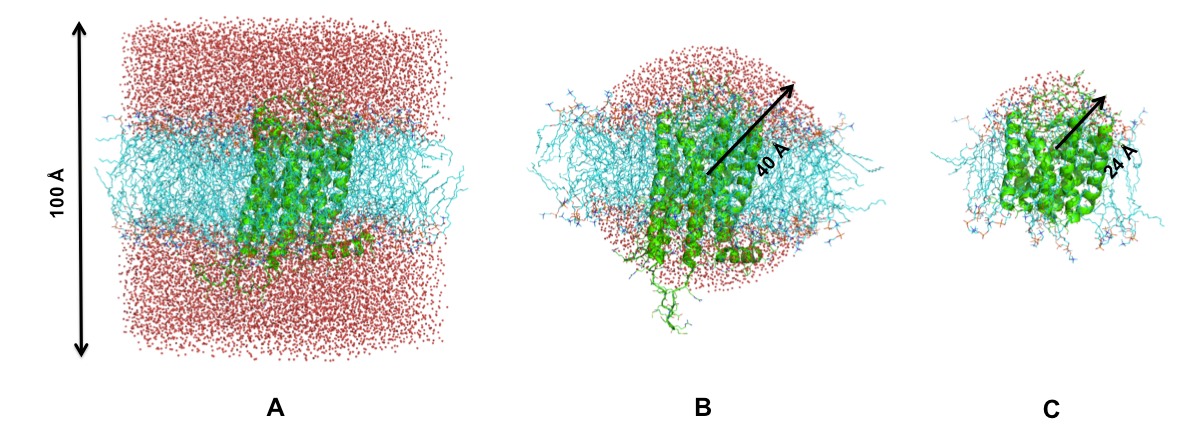

Supplement: Figure S2 — Schematic view of the three-step setup for the MD equilibration and production phases. (A) Starting model of the GPCR embedded in a lipid bilayer and simulated with PBC. (B) Equilibration of a 40 Å radius sphere centered on the ligand binding site that was cut out from the larger system. (C) The reduced model of the receptor-membrane-water system used for FEP calculations, where the radius of the simulation sphere is decreased to 24 Å. (TIF) [file pcbi.1003585.s002.tif]
