# Supplementary material for: Computational Prediction of Alanine Scanning and Ligand Binding Energetics in G-Protein Coupled Receptors
Source: PLoS Comput Biol. 2014 Apr 17;10(4):e1003585. doi: 10.1371/journal.pcbi.1003585 (PMC3990513; doi:10.1371/journal.pcbi.1003585)
Supplement: Table S1 — Ki values for BIBP3226 binding to wt and mutant hY1 from two different sources [15] , [16] . (DOCX) [file pcbi.1003585.s003.docx]

**Table S1.** ***K_i_* values for BIBP3226 binding to wt and mutant hY1 from two different sources** **[15,16].**

| Mutant | Sautel *K*_i_ [nM] | Sjödin *K*_i_ [nM] |
| --- | --- | --- |
| wt | 4.3 ± 0.5 | 7.6 ± 0.98 |
| D6.59A | >200 | - |
| F4.60A | >200 | 23 ± 1.0 |
| F6.58A | 21.7 ± 4.1 | - |
| N3.28A | - | 7.7 ± 0.5 |
| N6.55A | >200 | - |
| Q5.46A | >200 | - |
| S4.57A | 2.3 ± 1.4 | - |
| T5.39A | 7.5 ± 2.4 | 133 ± 12 |
| T6.52A | 1.3 ± 0.9 | - |
| T6.56A | 3.3 ± 0.3 | - |
| W6.48A | - | 153 ± 18 |
| Y2.64A | 6.6 ± 4.1 | - |
| Y5.38A | >200 | - |
