# Supplementary material for: Computational Prediction of Alanine Scanning and Ligand Binding Energetics in G-Protein Coupled Receptors
Source: PLoS Comput Biol. 2014 Apr 17;10(4):e1003585. doi: 10.1371/journal.pcbi.1003585 (PMC3990513; doi:10.1371/journal.pcbi.1003585)
Supplement: Table S2 — Structure and hY1 antagonistic activity of BIBP3226 analogs [17] , [18] . (DOCX) [file pcbi.1003585.s004.docx]

**Table S2.** **Structure and hY1 antagonistic activity of BIBP3226 analogs [17,18].**

| 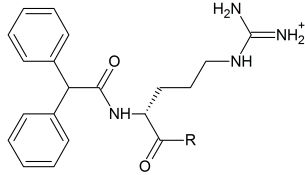 | | |
| --- | --- | --- |
| Compound*^a^* | R | hY1 antag.  IC_50_ [nM] |
| BIBP3226 | 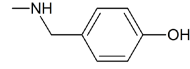 | 17 |
| **2** | 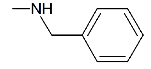 | 110 |
| **8** | 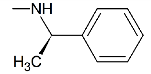 | 200 |
| **9** | 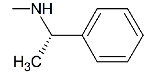 | 2500 |
| **11** | 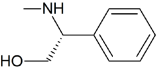 | 22300 |
| **12** | 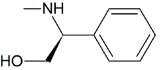 | 900 |
| **18** | 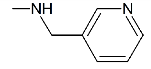 | 3750 |
| **25** | 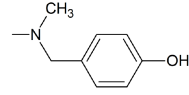 | 26 |

The ligand numbering is adopted from Aiglstorfer *et al.* [17,18].
