# Supplementary material for: Computational Prediction of Alanine Scanning and Ligand Binding Energetics in G-Protein Coupled Receptors
Source: PLoS Comput Biol. 2014 Apr 17;10(4):e1003585. doi: 10.1371/journal.pcbi.1003585 (PMC3990513; doi:10.1371/journal.pcbi.1003585)
Supplement: Table S3 — Calculated and experimental relative binding free energies of BIBP3226 analogs to wt hY1. (DOCX) [file pcbi.1003585.s005.docx]

**Table S3.** **Calculated and experimental relative binding free energies of BIBP3226 analogs to wt hY1**.

| Compound | ${\Delta G}_{holo}^{FEP}$ | ${\Delta G}_{wat}^{FEP}$ | ${\Delta\Delta G}_{FEP1}$ | ${\Delta\Delta G}_{FEP2}$ | ${\Delta\Delta G}_{FEP3}$ | ${\Delta\Delta G}_{FEP4}$ | ${\Delta\Delta G}_{FEP5}$ | ${\Delta\Delta G}_{bind}^{FEP}$ | ${\Delta\Delta G}_{bind}^{exp}$*^a^* |
| --- | --- | --- | --- | --- | --- | --- | --- | --- | --- |
| BIBP3226→**2** | 12.0 ± 0.6 | 11.1 ± 0.0 | -0.3 ± 0.6 | 0.0 ± 0.0 | 0.7 ± 0.1 | 0.6 ± 0.0 |  | 1.0 ± 0.6 | 1.1 |
| BIBP3226→**8** | 10.6 ± 0.5 | 11.0 ± 0.1 | -0.6 ± 0.3 | -1.0 ± 0.2 | 0.6 ± 0.1 | 0.6 ± 0.1 |  | -0.4 ± 0.5 | 1.5 |
| BIBP3226→**9** | 14.1 ± 0.4 | 11.2 ± 0.1 | 0.7 ± 0.3 | 0.5 ± 0.2 | 0.6 ± 0.1 | 1.1 ± 0.1 |  | 2.9 ± 0.4 | 3.0 |
| BIBP3226→**11** | 15.1 ± 0.3 | 8.8 ± 0.2 | 0.4 ± 0.1 | 0.4 ± 0.1 | 0.7 ± 0.1 | 4.7 ± 0.1 |  | 6.3 ± 0.3 | 4.3 |
| BIBP3226→**12** | 11.9 ± 0.5 | 8.3 ± 0.1 | -0.1 ± 0.3 | -1.4 ± 0.3 | 0.8 ± 0.0 | 4.4 ± 0.4 |  | 3.6 ± 0.5 | 2.3 |
| BIBP3226→**18** | -38.4 ± 0.7 | -39.9 ± 0.1 | 0.1 ± 0.4 | -0.1 ± 0.0 | 0.8 ± 0.0 | 0.7 ± 0.3 |  | 1.5 ± 0.7 | 3.2 |
| BIBP3226→**25** | 31.3 ± 0.7 | 30.0 ± 0.1 | -0.3 ± 0.4 | 0.3 ± 0.2 | 0.9 ± 0.3 | 0.3 ± 0.1 | 0.1 ± 0.1 | 1.4 ± 0.7 | 0.3 *^b^* |

The experimental values are estimated from IC_50_ values [17, 18]. Calculated energies (${\Delta\Delta G}_{bind}^{FEP}$) are obtained using a series of small, convergent FEP calculations (ΔΔG_FEP{X},holo_ and ΔΔG_FEP{X},wat_) and expressed in kcal/mol. *^a^* Experimental data from reference [18], except *^b^* data from reference [17].
